# Pathophysiology of age-related diseases

**DOI:** 10.1186/1742-4933-6-12

**Published:** 2009-09-08

**Authors:** Giuseppina Campisi, Martina Chiappelli, Massimo De Martinis, Vito Franco, Lia Ginaldi, Rosario Guiglia, Federico Licastro, Domenico Lio

**Affiliations:** 1 University of Palermo, Department of Oral Sciences, Palermo, Italy; 2 University of Bologna, Department of Experimental Pathology, Bologna, Italy; 3 University of L'Aquila, Department of Internal Medicine and Public Health, L'Aquila, Italy; 4 University of Palermo, Department of Human Pathology, Palermo, Italy; 5 University of Palermo, Department of Pathobiology and Biomedical Methodologies, Palermo, Italy

## Abstract

A Symposium regarding the *Pathophysiology of Successful and Unsuccessful Ageing *was held in Palermo, Italy on 7-8 April 2009. Three lectures from that Symposium by G. Campisi, L. Ginaldi and F. Licastro are here summarized. Ageing is a complex process which negatively impacts on the development of various bodily systems and its ability to function. A long life in a healthy, vigorous, youthful body has always been one of humanity's greatest dreams. Thus, a better understanding of the pathophysiology of age-related diseases is urgently required to improve our understanding of maintaining good health in the elderly and to program possible therapeutic intervention.

## Background

A Symposium regarding the Pathophysiology of Successful and Unsuccessful Ageing was held in Palermo, Italy on 7-8 April 2009. Three lectures from that Symposium by G. Campisi, L. Ginaldi and F. Licastro are here summarized. Elderly patients constitute a very heterogeneous group with a wide range of cultural, social and educational backgrounds. A worldwide phenomenon of the increasing number of elderly people has been recognized, with older people comprising a larger proportion of the population. This demographic phenomenon is related to an increase in chronic and at times debilitating diseases, associated with advancing age and an exponential growth in health costs. In some cases, a modification to life-style can reduce the so-called 'indirect' and 'intangible' costs of diseases in terms of public health. Thus, a better understanding of the pathophysiology of age-related diseases is urgently required to improve our understanding of maintaining good health in the elderly and to program possible therapeutic intervention.

## Section 1: Giuseppina Campisi and Rosario Guiglia - Systemic diseases in the elderly: relationships between hard and soft oral tissues

### Oral health in the elderly

The demographic changes described above could have a dramatic impact on mucosal and dental health: the elderly are at greater risk of oral diseases since gains in longevity result in more medically compromising conditions or systemic diseases, together with several oral-related manifestations.

Ageing affects oral tissues in addition to other parts of the human body, and oral health (the health of the mouth, teeth and associated structures, and their functional activity) is an integral component of general health. Indeed, oral diseases can have a profound impact on general health, as documented by the World Health Organization, whilst problems relating to general health can, and frequently do, manifest themselves in the mouth [1]. This relationship has been extensively treated in the literature, by scientific societies and Journals dedicated to this area of specialisation [2-4].

The most common oral conditions in the elderly are: dental caries, gingivitis, periodontitis, xerostomia, candidosis, denture stomatitis and oral cancer [5], while the most common systemic chronic diseases in this age group are arthritis and osteoporosis, Alzheimer's disease (AD), metabolic syndrome with diabetes mellitus, and cardiovascular diseases with myocardial infarction (MI). Furthermore, these latter conditions have potential sequelae in the oral district, particularly in older people and in medically-compromised adults. Finally, the treatment of these diseases with medication, chemotherapy, and radiotherapy has severe implications for the maintenance of oral health [6].

In such a context, it is worth noting that the relationship is not only in one direction (from ageing and systemic diseases towards to oral health), but more interestingly from the oral towards systemic diseases and potentially to unsuccessful ageing. Age-related oral changes are based on the same pathological dynamics as those generally recognised in all tissues: from tissue desiccation to diminished reparative ability, from reduced elasticity to altered cell permeability [7].

Older patients are more susceptible to root caries due to inadequate oral hygiene, infrequent dental examinations and cleaning, salivary gland dysfunction, an insufficient use of fluoride-containing oral hygiene products and removable partial dentures, which can trap plaque around the teeth and create an environment encouraging the formation of caries. External tooth changes include discoloration (from yellow to brown) and loss of enamel due to abrasion, erosion or occlusal attrition. Thinning around the neck of teeth, often related to the use of hard-bristled toothbrushes over many years of improper tooth brushing, is frequent [2]. Severe dental caries and periodontitis can lead to tooth extraction. Tooth loss impairs chewing, swallowing, and speaking, leading to nutritional deficiencies, social isolation, and depression [6].

Infections of the oral cavity constitute another important area in clinical dental practice. In elderly patients, atrophy of the oral mucosal epithelium occurs with aging but it may be more probably related to extrinsic factors (e.g. dentures) or disorders (e.g. vitamin B_12_/folate deficiency). This thinner and weaker oral mucosa may be more vulnerable to local irritation, resulting in a greater risk of candidal infection and/or reactivation of the varicella-zoster infection[6].

### Pathological Modification of oral flora in the elderly

Candidal infections are the most common of diseases due to an increased prevalence of salivary gland dysfunction, the use of removable prostheses, drugs that alter oral flora or the immune function (e.g. antibiotics, anti-neoplastic, corticosteroid and immuno-suppressing drugs), diabetes mellitus, malnutrition, and other immuno-compromising conditions. Oral candidosis may present itself as: a) acute pseudomembranous (commonly labeled 'thrush'), characterized by white plaques or patches that may cause pinpoint bleeding when scraped; b) being hyperplastic, with confluent leukoplakic plaques that cannot be scraped away; c) atrophic, characterized by painful erythematous mucosal lesions, frequently located beneath dentures; or d) angular cheilitis, characterized by leukoplakic and erosive lesions on the lip commissures [7,8].

Oral zoster is caused by the reactivation of a latent varicella-zoster virus infection. Precipitating factors include thermal, inflammatory, radiological or mechanical trauma, immuno-compromising states including cancer, Hodgkin's and non-Hodgkin's lymphoma, and physiological or emotional stress. Since these conditions are more prevalent in elderly people, herpes zoster is likely to an issue. Its appearance is characterized by a painful segmental eruption of small vesicles which rupture to form confluent ulcers. Vesicles appear on the skin and oral mucous membranes, occurring unilaterally along the ophthalmic, maxillary, or mandibular divisions of the trigeminal nerve. Post-herpetic neuralgia can last for months after the vesicles have erupted and cause considerable pain and neuralgia [9].

As a consequence of these infective events, dysphagia (i.e. difficulty in swallowing) or xerostomia can occur. The latter two are not considered as 'diseases' but conditions that give rise to a nutritional deficiency and reduced salivation respectively. Dysphagia and xerostomia in the elderly may also be caused by a variety of medical conditions, such as immunological (e.g. arthritis, diabetes), neurological (e.g. Parkinson's disease) or psychological disorders (e.g. depression, dementia), or by various types of medication (e.g. anticholinergic, antipsychotic, antihypertensive drugs) [10-12].

Oral cancer and potentially malignant lesions are concomitant with ageing. Indeed, exposure to oncogenetic factors (i.e. tobacco smoking, alcohol consumption, exposure to ultraviolet radiation, high risk human papilloma virus) as well as the inflammatory burden involved in onco-pathogenesis, increase during a person's lifetime [11] and could determine the occurrence of oral cancer precursors [13].

### Periodontal disease as a model of inflamm-aging

However, periodontal disease (PD) is, as a model of chronic inflammatory disease, able to influence the general status of health and the quality of ageing. PDs are a heterogeneous group of diseases that affect the supporting structures of the teeth (gingiva, root cement, alveolar bone and periodontal ligament). Generically, it is possible to distinguish gingivitis, as an early stage of the disease which does not involve tooth attachment and which displays irritated gums, from periodontitis, which affects all the tissue surrounding the tooth, concluding with regrettable dental loss. Its aetiology is complex, clinical manifestations are various and several classifications have been proposed. In 1999, the American Academy of Periodontology successfully classified PD in relation to its aetiology [14] in an International Workshop for the Classification of Periodontal Diseases and Conditions.

The chronic stimulation of inflammation sustained by the Gram-negative anaerobic bacteria of dental plaque has been correlated with various systemic diseases, such as pre-term and low birth- weight, atherosclerosis and cardiovascular diseases, worsening control over diabetes and the slow healing of wounds, aspiration pneumonia and osteoporosis [15].

Different models of the aetiopathogenetic mechanisms of oral bacteria exist:

1) Common susceptibility involves a genetically determined phenotype. In the presence of periodontal pathogens, a susceptible patient develops PD. This same person would also be susceptible to atherosclerosis, diabetes or pulmonary infections but, in this model, PD does not cause a systemic condition;

2) at present, a more complex model is accredited, in which direct and indirect dynamics as well as innate, endogenous and exogenous factors are involved. Local adaptive immunity reacts to produce cytokines which are capable of altering vessel permeability, thereby enabling monocytes to penetrate the inflamed tissue [16]. The chronic stimulation of inflammation by bacterial plaque involves several cell populations and several networks of cytokines, facilitating detachment and the formation of bone defects, since the cell populations amplify the inflammatory reaction and activate the effector mechanism, which is responsible for tissue destruction. At the same time, bacteria reaches the blood circulation directly or by means of their lipopolysaccharides;

3) this model offers the basic rationale for the documented link between local effects and various related systemic diseases. Specifically, a bi-directional relationship has been reported to exist for many diseases, the latter which could be reciprocally influenced [15]. Of these, diabetes is one of the most reported diseases with a very precise pathological dynamic: the higher susceptibility of diabetics to periodontitis. The latter enhances insulin resistance and complicates glycaemic control, in addition to determining a more severe case of periodontitis, leading to defining periodontitis as the sixth complication of diabetes.

The second bidirectional condition is the presence of osteoporosis (primary and secondary one), which is correlated with the loss of alveolar bone and teeth. Hence, osteoporosis involves the same percentage of the stomatognathic apparatus as with the skeleton, and in numerous studies it has been correlated with a loss of alveolar bone and teeth [17,18] (see the next paragraph). This model had previously been established in relation to unidirectional pathogenesis (from osteoporosis forward to PD), but today the same dynamics for osteoporosis have also been hypothesised as for diabetes, based on the release of cytokines at the onset of PD. The latter induces an uncoupling of normal bone homeostasis, an increase in osteoclastic activity and decrease in bone mineral density.

Recently new links have been hypothesized between PD and renal disease, obesity, dysmetabolic syndrome and pancreatic cancer. However, possibly the most interesting link suggested to date is that with Alzheimer's disease (AD) [19], a chronic age-related disease depending on systemic and local inflammation [18]. The invasion of the brain by oral bacteria was posited as recently as 2002 [20]: of the periodontal bacteria, various species such as *Actinobacillus Actinomycetemcomitans, Porphyromonas gingivalis, Treponema denticola *and *Fusobacterium nucleatum *have been found to be capable of invading the brain, modifying the cytokine milieu and possibly contributing to existing pathological mechanisms. In particular, *Treponema species*, including *T. denticola*, have been detected in 14/16 AD and 4/18 non-AD brains. Moreover, AD specimens have displayed a greater number of *Treponema species *than the controls [20].

Two mechanisms may be involved in the PD-induced onset/progression of AD:

1) inflammatory; and 2) bacterial mechanisms. The first mechanism implies that PD-derived inflammatory molecules increase brain inflammation. The interaction between periodontal bacteria and host response results in the locally increased production of inflammatory molecules, including interleukin (IL)-1β, IL-6, IL-8, Tumor Necrosis Factor (TNF)-α, and C-reactive protein (CRP).

The host response to subgingival periodontal pathogens engages both innate and instructive immune responses, resulting in the alteration of local vasculature, generation of an inflammatory response, immune cell priming, and the secretion of pro-inflammatory cytokines. The bacteria and host response of a patient in good periodontal health are in equilibrium. In cases of gingivitis, the bacterial challenge elicits an innate immune response in the adjacent gingival tissue, which is capable of limiting a bacterial-induced pathology. When periodontitis has been diagnosed, the balance between bacteria and host response is disrupted, thereby resulting in an increased inflammatory infiltrate and the production of pro-inflammatory cytokines. Tissue destruction occurs mainly by the activation of osteoclasts, matrix metalloproteinases, and other proteinases by the host inflammatory response.

In cases of severe PD, these pro-inflammatory molecules may induce a systemic inflammation and may, therefore, access the brain via systemic circulation. Pro-inflammatory molecules, derived locally from periodontal tissue, may stimulate trigeminal nerve fibers, leading to an increase in the number of brain cytokines [21]. These cytokines may act on the already primed glial cells, resulting in an amplified reaction and possible progression of AD. A test for this hypothesis would entail examining whether PD affects the progression of AD, which clinically presents as earlier onset or as a more severe stage of the disease.

The second mechanism by which PD could contribute to brain inflammation is direct, through bacteria and/or bacterial products. Several bacteria, including oral bacteria, have been hypothesized as being implicated in the pathogenesis of AD [22]. The mechanism by which periodontal bacteria access the brain is unknown. However, the mechanisms described for other bacteria accessing the brain via systemic circulation is possible. Orally-originated bacteriaemia occurs relatively frequently during dental and non-dental manipulations. A further route by which bacteria may reach the brain is via the peripheral nerves. Riviere' studies have demonstrated that spirochete *species *were detected in the trigeminal ganglia, thereby suggesting the ability of oral spirochetes to invade the central nervous system via the peripheral nerves [20]. However, the simple presence of periodontal bacteria in the systemic circulation or in the territory of peripheral nerve fibers does not imply access to the brain. Additional co-factors may be required, such as age, the presence of inflammatory cytokines or other infections [23,24]. Although it is most probable that infections are not causative in these types of diseases, their possible role as aggravating co-factors in patients with susceptible genetic backgrounds should be seriously considered. Consequently, every chronic peripheral infection may contribute to the global infectious/inflammatory burden and participate in the aetiopathogenesis of relevant diseases.

Several related cross-sectional studies [25-29] and various longitudinal studies [30,31] have demonstrated that patients with dementia are more likely to have poor oral health. For example, the Nun Study, a longitudinal study of ageing and AD, provided an opportunity to study oral health and cognitive function. The authors of this study confirmed that fewer teeth increased the risk of a higher prevalence and incidence of dementia (also in patients without the Apo-E4 allele), albeit without conclusive evidence regarding the 'causal or casual' role played by each factor [32].

In conclusion, evidence-based data regarding the association of PD with neurodegenerative disorders are still lacking. However, it seems plausible that there is an increase in the number of brain cytokines activating the neurodegenerative pathway via a contribution to systemic/brain inflammation.

## Section 2: Lia Ginaldi - Osteoporosis and immunosenescence

Osteoporosis is an age-related disorder, representing a major cause of morbidity and mortality in older people, together with other age-related diseases, such as atherosclerosis, tumors, metabolic syndrome and neurodegenerative disorders. It is a systemic pathology of the skeleton characterized by loss of bone mass, decreased bone mineral density and loss of microarchitectural integrity leading to increased fragility and consequent risk of fractures. Osteoporotic bone is demineralised but also modified in its architectural structure with trabecular disruption and resistance loss. Therefore, osteoporosis is fundamentally an asymptomatic condition until the appearance of a bone fracture presenting itself as a complication with clinical visibility and often lifethreatening [32]. Everything before the fracture has remained long unknown and it is only recently that the emerging discipline of osteoimmunology is clarifying its pathogenesis by providing a new reading register of senile osteoporosis in the light of immunosenescence and inflamm-ageing.

Immunosenescence is the consequence of the continuous attrition caused by lifelong antigenic load which is responsible for the chronic immune system activation and hyperproduction of pro-inflammatory cytokines. Inflamm-ageing is the condition of chronic inflammation characterizing ageing. It is the consequence of the capacity of the immune system to counteract stress agents and represents the background underlying a wide range of age-related diseases which share an inflammatory pathogenesis [33].

Ageing and oestrogen deficiency are the two most important risk factors in developing osteoporosis. Osteoporosis was in fact firstly described as progressive lightness and softness of bones acquired during ageing. Later postmenopausal osteoporosis was defined as a consequence of oestrogen deficiency. Currently osteoporosis is viewed as a heterogeneous condition which can occur in any age of life and its aetiology is attributed to various endocrine, metabolic and mechanical factors. Recently, growing understanding of bone physiology suggests that factors involved in inflammation are linked with those critical for bone remodelling process, supporting the theory that immunosenescence significantly contributes to the aetiopathogenesis of osteoporosis. Therefore senile osteoporosis could be considered as an immune mediated disease or at least the result of an inflammatory process [34].

In the postmenopausal period there is coincidence of inflammation with osteoporosis. Immunological dysfunctions, autoimmune and chronic inflammatory diseases, HIV infection, rheumatic disorders, such as rheumatoid arthritis, and lymphoid neoplastic diseases, are associated with osteoporosis. Erosions seen in conditions such as rheumatoid arthritis, ankylosing spondylitis, and psoriatic arthritis, are typically associated with inflammation in the joints. Inflammation and bone reabsorption share several soluble mediators, such as IL-1, IL-6 and TNF-α. There is an inverse correlation between levels of CRP, a reliable marker of systemic inflammation, and bone mineral density [35]. Particularly interesting, although less immediately evident, is the link between immunity and osteoporosis in advanced age, in which other well-known causes of bone reabsorption are also present. However, a careful reading of osteoporosis reveals how the peculiar age-related immune system remodellingitself represents the most important pathogenesis factor also for senile osteoporosis.

### Immune-mechanisms of the osteoporosis

The skeleton is physiologically in a state of dynamic equilibrium between new bone formation mediated by osteoblasts and reabsorption mediated by osteoclasts. Both these processes are finely tuned by cytokines and growth factors. Dendritic cells, specialized to present antigens, and osteoclasts, specialized to reabsorb bone, share the same bone marrow precursors of the monocyte lineage and exhibit parallel lifecycles, regulated by a variety of cytokines. Release of cells into the circulation from the bone marrow and homing from the blood stream to peripheral tissues where the immature osteoclast precursors differentiate into mature osteoclasts are complicated processes involving adhesion molecules, cytokines and chemokines. They differentiate into CD11c+ dendritic cells in the presence of GM-Colony stimulating factor(CSF) plus IL-4 but form TRAP+ osteoclasts if exposed to receptor activator of nuclear factor kappa B (RANK)-ligand(L) and M-CSF. The dendritic cells produce cytokines and chemokines directly or activate T lymphocytes to indirectly promote osteoclasts and inflammation [36].

The main sìgnalling pathway in bone reabsorption is mediated by the stimulation of RANK receptor on osteoclasts and their precursors by its specific RANK-L, predominantly expressed on osteoblasts and stromal cells. This receptor system pertains to TNF-family molecules and is essential for the development and activation of osteoclasts. A central role in this system is also played by the ligand osteoprotegerin (OPG), competitive inhibitor of RANK-L, also known as osteoclastogenesis inhibitory factor, which functions as a soluble decoy receptor to RANK-L. Inhibition of RANK-L function via OPG prevents bone loss. Other costimulatory immune receptors also exist, which act cooperatively with RANK-L in enhancing osteoclastogenesis. In the immune system, RANK-L is expressed by activated T cells, B cells and dendritic cells. Therefore activated T lymphocytes could directly induce osteoclastogenesis through RANK-L. Following antigen recognition, T cells become activated and produce RANK-L that induce osteoclast differentiation and activation. Both these processes could be downregulated by the decoy receptor OPG. In addition, they produce inflammatory cytokines, such as tumour necrosis factor, interleukin-1, interleukin-6, which induce osteoblasts to further express RANK-L [37]. All of these lead to an imbalance between bone formation and reabsorption, with consequent osteoporosis. Therefore it is the activated immune profile which, through inflammation and inflammatory cytokine production, modulates osteoblast and osteoclast activity leading to osteoporosis.

That's exactly what happens in inflamm-ageing in which maintenance and amplification of inflammatory reaction leads to osteoclastogenesis and increased risk of fractures. The inducer cells in this process are immune cells such as activated macrophages and lymphocytes, which produce cytokines and soluble mediators able to stimulate osteoclast differentiation and activation. Therefore osteoporosis and immunosenescence share the same immunological cell and cytokine mediators. Ageing is accompanied by increased TNF-α, IL-1, RANK-L and M-CSF expression and expansion of the osteoclast precursor pool. As a consequence, there is an increased stromal/osteoblastic cell-induced osteoclastogenesis during ageing. The increased production of pro-inflammatory cytokines with ageing derives from a chronic hyperactivation of macrophages and dendritic cells, as well as memory/effector and senescent T cells. These cytokines induce expansion of osteoclast precursors which in turn may contribute to the maintenance of inflammation through their capability to produce proinflammatory cytokines themselves and recruit other inflammatory cells, rendering the inflammation chronic and inducing osteoporosis [38,39]. Characteristic of an aged immune profile is the accumulation of activated memory/effector cells expressing RANK-L, preferentially resident in the bone and secreting osteoclastogenic pro-inflammatory cytokines [40].

Thanks to the most recent acquisition in the field of osteoimmunology, new drugs for osteoporosis therapy have been synthetized, the so-called biological drugs. They are specifically able to inhibit the RANK/RANK-L activation signal with consequent blocking of osteoclast differentiation, activation and survival. These new drugs are recombinant OPG and RANK receptor, which competitively block RANK-L ligand and do not allow the interaction with its receptors, and above all human monoclonal antibody anti-RANK-L, denosumab. The latter specifically inhibits the function of its target through immunological recognition. The therapy with these monoclonal antibodies (MoAbs) subcutaneously administered every 3-6 months is already in the advanced experimental clinical stage in postmenopausal osteoporotic women for verifying the anti fractural efficacy and long-term systemic and bone effects. The preliminary results from these trials appear promising [41]. Finally, human MoAbs anti-RANK-L could also be useful in the prevention and therapy of bone metastasis of osteolytic tumor, mainly breast cancer and myeloma. The osteoclastic bone re-absorption, a well known tumor complication, also favors itself neoplastic cell burden in skeleton. Therefore RANK-L blockage, in addition to inhibit bone re-absorption, also reduces tumor growth, increases apoptosis of malignant cells and reduces proneoplastic inflammation [42].

## Section 3: Federico Licastro and Martina Chiappelli - Gene epistasis, interaction between gene and environmental risk factors in chronic age-related diseases

### Genetic polymorphisms and age related diseases

Recognition and treatment of established risk factors, for example of MI, have considerably reduced the disease burden. However, phenotypic markers vary over time and their predictive potential is affected by age, gender, diet, co-morbidity, drug treatment and other environmental variables. Moreover, the relevance of these variables on the atherosclerosis progression and clinical manifestation of MI might be also differentially influenced by the individual genetic background [43]. Taking into account theses figures, it is clinically relevant to develop new risk chart and statistical algorithm for cardiovascular diseases that comprise genetic risk factors and their interaction with other clinical and epidemiological interaction.

Treatment for other age-related diseases, such as AD, is up today symptomatic and no therapeutic treatment, so far tested, has been able to cure or slow down the clinical progression of dementia. Therefore, developing a risk chart for AD and cognitive decline is a very important goal to reach early intervention therapy and possibly prevention of age related cognitive decline and dementia [44].

Assessment of genetic risk factors might help in better defining individual risk or predisposition to MI or AD. [43,44]. In fact, inherited gene variants are less influenced by environmental factors and might provide a better indication for individual susceptibility to these diseases. Moreover, efforts for unraveling the genetic basis of MI and AD are pivotal for the development of new diagnostic tools and innovative therapeutic approaches. Multiple pathogenetic pathways leading to MI or AD implicate genetic heterogeneity, or, in other words, the association of multiple genetic traits with the disease. For instance, plasma lipoprotein levels may be influenced by several genes regulating different metabolic pathways. Transition from stable to unstable atherosclerotic plaque may be affected by several gene polymorphism in different genes with immune regulatory functions. Moreover, a single gene polymorphism (SNP) can account for a limited contribution (low odds ratio values) to the total genetic load for the disease and both common and rare gene polymorphisms may differentially influence the susceptibility to the disease. On the other hand, the presence of one or more established risk factors for MI or AD might differentially influence gene expression and in turn the clinical relevance of one or more genes associated with the disease. The above notions may partially explain contradictory results of genetic association studies from MI and AD [45-48]. Therefore, investigations designed to evaluate gene-gene and gene-environment interactions for identification of a multi variable network associated with increased risk of developing AMI or AD might be highly informative.

### The statistical approach to the identification of risk for multifactorial diseases

The statistical evaluation of multiple variables in a sufficiently large population is another complex issue and new statistical models able to connect several factors with the disease, to evaluate the degree of linkage among variables and their association with the disease or its absence are needed. The more common algorithms of linear projections of variables are the principal component analysis (PCA) and the independent component analysis (ICA); the former requires a Gaussian distribution of data, while the latter does not require any specific distribution. These classical statistical techniques have limited power when the relationships between variables are non linear. Moreover, PCA and ICA are not able to preserve the geometrical structure of the original space. Application of these methods may lose important information and establishing precise association among variables having only the contiguity as a known element is difficult. Another limitation of currently used statistical methods is that mapping is generally based on a specific kind of "distance" among variables (e.g. Euclidean, City block, correlation, etc) and gives origin to a "static" projection of possible associations. In other words, the intrinsic dynamics due to active interactions of variables in living systems of the real world (which could be captured by means of artificial adaptive systems) is completely lost. A connection scheme able to hypothesize links among variables, i.e. minimum spanning tree (MST) algorithm, as described by Kruskal (1956) [49], could increase the information obtained by the map. The Kruskal MST algorithm of graph theory finds a minimum spanning tree for a connected weighted graph. MST method finds a subset of the edges that form a tree that includes every vertex, where the total weight of all the edges in the tree is minimized. This function has been recently applied in the medical field, especially in biology and medical imaging. However, the MST algorithm is still rare in medical clinics.

To evaluate the relationship among genetic, clinical traits and classical risk factors in these diseases a new statistical model based upon the Auto Contractive Map algorithm (AutoCM) has been applied. This novel data mining AutoCM algorithm [50] was aimed to explore the concomitant association of different variables with MI or AD and the potential relationships among variables in a multi factor network relevant for the disease. This novel data mining algorithm was aimed to explore the concomitant association of different variables with AD and the potential relationships among variables in a multi factor network relevant for the disease. The ultimate goal of this data mining model was to discover hidden trends and associations among variables, since this algorithm was able to create a semantic connectivity map in which non linear association were preserved and explicit connection schemes were described [50]. This approach describes a context typical of living systems where a continuous time dependent complex change in the variable value is present. After the training phase, the matrix of the AutoCM represents the warped landscape of the dataset. A simple filter (minimum spanning tree by Kruskal) to the matrix of AutoCM system was introduced; this approach shows the map of relevant connections between and among variables and the principal hubs of the system. Hubs can be defined as variables with the maximum amount of connectivity in the map.

### Auto Contractive Map algorithm and evaluation of and environmental risk factors in chronic age-related diseases

To evaluate the relationship among genetic, clinical traits and classical risk factors in these diseases a new statistical model based upon the Auto Contractive Map algorithm (AutoCM) has been applied. This novel data mining AutoCM algorithm [50] was aimed to explore the concomitant association of different variables with MI or AD and the potential relationships among variables in a multi factor network relevant for the disease. The ultimate goal of this data mining model was to discover hidden trends and associations among variables, since this algorithm was able to create a semantic connectivity map in which non linear association were preserved and explicit connection schemes were described [51]. This analysis showed an age and gender dependent hierarchy of partially diverse biological factors with these diseases.

Results from this investigation showed that blood cholesterol levels, a polymorphism in the hydroxyl-methyl-glutaril-CoA-reductase gene (HMGCR), the limiting enzyme controlling cholesterol synthesis, and age were the principal risk factors associated with AD and vascular dementia. Others inflammatory gene and blood levels of the cognate molecules were differently linked to dementia by the above mentioned main hubs in the connectivity map for the disease.

When applied to MI case control study this analysis showed that several polymorphism of inflammatory genes such as IL-6, VEGF, IL-1beta, and cholesterol metabolism i.e. HMGCR gene, low blood HDL and diabetes were directly linked to the risk of developing MI at early ages (< 50 years). These new finding potentially describe a new risk chart for MI by integrating age, classical risk factors, and new genetic risk factors.

In conclusion, this new statistical analysis showed a connectivity map among variables overall describing an age and gender dependent interaction of different gene variants with AD or MI [51,52]. Our findings suggest that the concomitant assessment of multiple variable relationship with complex diseases by powerful algorithm may results in a new data mining method to apply in the developing field of predictive diagnostics (Licastro F, Chiappelli M, Porcellini E, Campo G, Buscema M, Gross E, Garoia F, Ferrari R: Gene-gene and gene-clinical factors interactions in acute myocardial infarction. submitted.). Moreover, this method suggests a road leading to an innovative risk evaluation map that, by including genetic factors, integrates already established risk charts to evaluate individual predisposition to MI or AD.

## Section 4: Vito Franco and Domenico Lio - Conclusion and Final remarks

Age-related diseases constitute a heterogeneous complex, ranging from entities of little impact on general health but important for psychological and social lives, to severe illness both life-threatening or leading to permanent disability. The study of pathophysiology of these conditions has significantly contributed to better understanding of underlying mechanism along with important suggestions for possible therapeutic targets. Therefore, the effort to allocate resources in the study of ageing cannot be considered minor in respect to other leading fields such as oncology, cardiology, etc. Topics treated in this section are strictly related each-one considering that the Developed Country life style have increased the post reproduction age life time with a significant prolonged time interval that should be defined aging. This is a complex trait dealing only in part with the evolutionary programmed human life span expectancy. Genes and systems adapted to assure the specie successful expansion may be detrimental, at individual level, for successful ageing. Immune system and inflammation seem to be the better examples of these contrasting effects.

### Oral infections, antigenic burden and inflamm-aging

Actually bacterial flora colonise all mucosal surfaces at least one month after birth and the innate immune system through a low grade of inflammation controls the pathogenetic potential of bacteria in particular in the mouth cavity. As well discussed in the first section of the report by Giuseppina Campisi the increased antigen burden associated to oral diseases in aging, e.g. dental caries, gingivitis, periodontitis, inducing a chronic inflammatory stimulation increase the risk for the main aging associated diseases as atherosclerosis and Alzheimer diseases.

### Inflamm-aging and osteoporosis

Antigenic load which is responsible for the chronic immune system activation and hyper production of pro-inflammatory cytokines seems to play a role in osteoporosis. As discussed by Lia Ginaldi (see above) growing understanding of bone physiology suggests that factors involved in inflammation are linked with those critical for bone remodelling process, supporting the view that inflamm-aging significantly contributes to the aetiopathogenesis of osteoporosis.

### Analysis of the interactions among genes implied in inflamm-aging and age-related diseases: Advanced statistical models to study epigenetic interactions

In this scenario polymorphisms of genes involved in inflammation or immune response that can be protective in the first ages of the life facilitating both immune and inflammatory response might result detrimental in the elderly [53]. So it is very difficult to individuate genetic profiles that, interacting with clinical traits and environmental and life style risk factors, might allow to identify individuals with a major risk for one or more aging related diseases. Actually the epigenetic interaction might be not evidenced by the formal statistic models as multiple regression or likelihood algorithms. Advanced statistical models as the Auto Contractive Map algorithm (AutoCM) presented by Federico Licastro might be more powerful even if, probably, model based on neural networks able to elaborate more than one thousands variables should be necessary. "Artificial Intelligence" might allow to find parameters and markers hidden in the mass of the data collected that might be useful for prediction and prevention strategies and for health policy applications at large strata of population against ageing related diseases [54] and to reach a successful ageing characterised by the better quality rather than quantity in life span expectation.

## Competing interests

The authors declare that they have no competing interests.

## Authors' contributions

All the Authors drafted the manuscript and approved the final manuscript.
